# Prognostic value of CA 19-9 levels in patients with inoperable adenocarcinoma of the pancreas treated with gemcitabine

**DOI:** 10.1038/sj.bjc.6601263

**Published:** 2003-10-14

**Authors:** C Ziske, C Schlie, M Gorschlüter, A Glasmacher, U Mey, J Strehl, T Sauerbruch, I G H Schmidt-Wolf

**Affiliations:** 1 Medizinische Klinik und Poliklinik I, Rheinische Friedrich-Wilhelms-Universität, Sigmund-Freud-Str. 25, 53105 Bonn, Germany

**Keywords:** Ca19-9, pancreatic cancer, gemcitabine

## Abstract

Serum carbohydrate antigen 19-9 (CA 19-9) has been identified as a useful tumour marker for diagnosis of exocrine pancreatic carcinoma, but its value for evaluating the response to chemotherapy with gemcitabine is not clear. Tumour regression in pancreatic carcinoma is hard to determine due to massive desmoplastic tissue. Furthermore, objective tumour response does not automatically transcribe into better survival. Therefore, clinical benefit response, a composed parameter consisting of factors like performance status, pain, and body weight was integrated in evaluating tumour response. The aim of this prospective study was to evaluate the usefulness of serial CA 19-9 measurements as a biochemical response marker and an outcome prognostic parameter in patients with advanced pancreatic cancer receiving gemcitabine treatment. A total of 46 consecutive patients (median age 66 years) suffering from histologically proven locally advanced or metastatic adenocarcinoma of the exocrine pancreas were analysed. Gemcitabine was applied for a median of 23 courses (range 6–76). Two patients achieved an objective complete remission, five an objective partial remission (overall response, OR=15.2%), while objective stable disease was documented in 19 and objective progressive disease in 20 patients. Patients with a decrease of >20% of the baseline CA 19-9 level after 8 weeks of chemotherapy had a significantly better median survival than patients with a rise or a decline <20%. The response of CA 19-9 >20% during chemotherapy was the only independent predictor of survival in a multivariate analyses. In contrast, neither objective tumour response nor clinical benefit response showed this level of significance. In conclusion, kinetics of CA19-9 serum concentration serves as an early indicator of response to gemcitabine chemotherapy in advanced pancreatic cancer.

Pancreatic cancer is predominantly diagnosed in advanced stages of the disease. In all, 80–95% of patients are inoperable at diagnosis and only about 1% of patients are still alive 5 years from the time of diagnosis (Ahlgren, 1996). Gemcitabine (2′,2′di-fluorodeoxycytidine), a nucleoside analogue with a mild toxicity profile (Aapro *et al*, 1998), has been shown to improve both clinical benefit and survival in patients with advanced pancreatic cancer compared to treatment with 5-fluorouracil, although overall survival was poor with a median survival of less than 6 months (Burris *et al*, 1997). Especially, in pancreatic cancer a common problem with chemotherapeutic approaches consists in the difficulty in determining objective treatment response (Warshaw and Fernandez-del Castillo, 1992; Rothenberg *et al*, 1996a). This is partly due to the retroperitoneal localisation of the pancreas, and also due to the limited possibilities of differentiating the tumour from the normal surrounding tissue. Moreover, inclusion of desmoplastic tissue into the baseline tumour volume may cause an underestimation of tumour reduction during chemotherapy, while inclusion of surrounding inflammatory tissue may result in an overestimation of response (Rothenberg *et al*, 1996a). Therefore, great efforts have been undertaken to determine the impact of chemotherapy by methods other than imaging of tumour volume (Rothenberg *et al*, 1996a; Burris *et al*, 1997). One approach has been the measurement of clinical benefit that is a composed parameter consisting of factors like performance status, pain, and body weight. While an evaluation of clinical benefit response reflects on the quality of life, this approach is time consuming, in many aspects subjective, and certainly controversial in its validity (Burris *et al*, 1997). In patients undergoing radiation or resection of pancreatic cancer, the prognostic value of serum carbohydrate antigen 19-9 (CA 19-9) has already been accepted. In search for a parameter reflecting the chemotherapeutic efficacy faster, on a more objective level, and to identify any subgroups of patients in whom chemotherapy with gemcitabine improves survival, prognostic parameters (tumour grading, tumour stage, prior palliative operation, baseline CA 19-9 and CA 19-9 decrease) during the first treatment courses, CA 19-9 was evaluated in patients undergoing chemotherapy with gemcitabine alone (Halm *et al*, 2000) or in combination with cisplatin (Heinemann *et al*, 1999). Halm *et al* (2000) and Heinemann *et al* (1999) found that kinetics of CA 19-9 concentration may serve as an early indicator of response to gemcitabine/gemcitabine–cisplatin therapy. Serum carbohydrate antigen 19-9, the sialylated Lewis blood group antigen defined by the monoclonal antibody 1116 NS 19-9 (Koprowski *et al*, 1979), is a tumour-associated antigen synthesised by normal pancreatic and ductular cells occurring in large quantities in normal pancreatic juice (Steinberg, 1990). It is the most sensitive and specific serum marker for pancreatic cancer (Pleskow *et al*, 1989; Rollhauser and Steinberg, 1998). The prognostic value of CA 19-9 for patients with pancreatic cancer treated by resection or radiotherapy is well established (Glenn *et al*, 1988; Katz *et al*, 1998; Rollhauser and Steinberg, 1998), but only few data regarding the prognostic value of CA 19-9 during chemotherapy with gemcitabine have been published so far. No prospective studies comparing clinical benefit response and CA 19-9 response were performed. Although CA 19-9 is not pathognomonic of pancreatic cancer, most patients show elevated serum concentrations of this marker. This study analyses the kinetics and the prognostic value of the tumour marker CA 19-9 as a biochemical response marker before and during chemotherapy in patients with advanced or metastatic pancreatic cancer treated with gemcitabine with special emphasis on objective tumour and clinical benefit response.

## PATIENTS AND METHODS

A total of 46 consecutive patients with a baseline Karnofsky performance status ⩾60%, 20 female and 26 male patients at a median age of 66 years (range 38–85 years) who had not received prior treatment were evaluated. All patients suffered from inoperable adenocarcinoma of the exocrine pancreas. In total, 13 patients had stage III disease, and further 33 patients had stage IV disease. Liver metastases were observed in 31 patients. For remission evaluation, imaging techniques including ultrasound, computerised tomography (CT)-scan, or magnetic resonance tomography (MRT) were used at intervals of one chemotherapy treatment course. Only bidimensionally measurable lesions were evaluated.

### Chemotherapy regimen

All patients received chemotherapy with gemcitabine as a single-agent therapy as described previously (Rothenberg *et al*, 1996b; Burris *et al*, 1997). During the first treatment course, patients received gemcitabine at a dose of 1000 mg m^−2^ once weekly for 7 weeks followed by 1 week of rest (first treatment course). Thereafter, gemcitabine was given once weekly for 3 weeks followed by 1 week (second and following treatment course) of rest until progression of disease. Assessments of objective tumour response were made following standard WHO criteria (attachment 4) (WHO, 1979). CT scan was applied after the first treatment course (i.e. after 8 weeks) and after every second treatment course (i.e. after additional 8 weeks). Progression was defined either by an increase of >25% of the longest perpendicular diameters of a mass lesion, the occurrence of a new lesion or malignant ascites, or a deterioration of performance status with inability of the patient to attend follow-up visits.

### Assessment of clinical benefit response

Clinical benefit response is a measure of symptomatic improvement based on the following three most common debilitating signs or symptoms present in patients with advanced pancreatic cancer: level of pain, consumption of pain medication; ability to perform daily activities; weight change. Subjects were considered to be clinical benefit responders according to previously described criteria (Rothenberg *et al*, 1996a) only if they experienced improvement in at least one measurement without deterioration in any of the others. This improvement had to reach an established level for at least 4 consecutive weeks. Pain was assessed by pain intensity and analgesic consumption, functional impairment, assessed by Karnofsky Performance Status (KPS), and weight loss every 4 weeks.

### Determination of CA 19-9 serum concentration

Serum samples for determination of the tumour marker CA 19-9 were obtained from every patient at baseline, after the first three courses (3 weeks) and after completion of the first treatment course (8 weeks) and thereafter before each treatment course (i.e. every 8 weeks). In case of stent obstruction during chemotherapy, CA 19-9 measurements were excluded from the evaluation and after exchange of the stent was performed serum samples were obtained again. The CA 19-9 concentration was measured by an automated, commercially available chemiluminescent immunoassay (GI-MA CA 19-9, Corporation Immulite 2000, Bad Nauheium, Germany). A value of 20 U ml^−1^ was used as the upper limit of normal. The coefficient of variation in our laboratory was 6.5% (*n*=55). Taking the coefficient of variation into account, a decrease in CA 19-9 during chemotherapy was defined as a decrease >20% after the first treatment course (8 weeks) of chemotherapy (i.e. biochemical response).

### Statistical analysis

Unadjusted median survival curves were plotted according to Kaplan and Meier (1958). Differences in survival were calculated with the log-rank test and correlations with Spearman's rank correlation test. For the univariate survival rate analysis, the overall survival time (defined as the time between first diagnosis and death by any cause) was used as a follow-up parameter. Cox univariate regression analysis was used in the analysis of the associations between the different variables and overall survival (Cox, 1972). Two-sided *P*-values below 0.05 were considered to be statistically significant. All computations were performed with the Statistical Package for Social Sciences (SPSS), Version 10.0 (SPSS Inc., Chicago, IL, USA).

## RESULTS

Patient characteristics are listed in Table 1

**Table 1:** Categorical distributions of baseline characteristics in all patients

**Patient characteristics**	
Age (years, range)	66 (38–85 years)
Males/females	26/20
Median baseline CA 19-9 (U l^−1^)	384
	(range 18–9578 U l^−1^)
*Tumour stage (UICC 1997)*	
III/IV A	13
IV B	33
*Tumour grading*	
I	2
II	28
III	8
IV	8
Biliary prosthesis	16
*Clinical benefit response*	
Improved	12
Not improved	34

. A total of 46 patients with advanced pancreatic cancer were evaluated consecutively. The median age was 66 years (range 38–85 years), 26 patients were male and 20 were female. In all, 13 patients had stage III/IVA disease, while further 33 patients had stage IVB disease. Liver metastases were observed in 31 patients. In total, 16 patients with obstructive jaundice underwent endoscopic biliary prosthesis prior to chemotherapy. Two patients had undergone palliative operation; no patient had previous Whipple's pancreatoduodenectomy. Most patients had moderately or poorly differentiated ductal adenocarcinoma. A median number of 23 chemotherapy infusions (range 6–76) were applied. In all, 28 patients with progressive disease received a second-line chemotherapy with gemcitabine (1000 mg m^−2^) administered i.v. d 1, 8, 15 and 22 followed by a 3-week rest period and folic acid (200 mg m^−2^), administered i.v. as a 30 min infusion following gemcitabine administration, followed by 5-FU (750 mg m^−2^) as a continuous 24-h infusion on days 1, 8, 15, 22.

In 82% of the gemcitabine administrations, doses were given on schedule. Dose reduction, mostly related due to haematological toxicity grade 3 during treatment courses, was performed according to the manufacturer's proposals.

Two patients achieved an objective complete remission, and five achieved an objective partial remission (OR=15.2%). Objective stable disease (⩾2 months) was documented in 19 patients and objective progressive disease in 20 patients. Serum carbohydrate antigen 19-9 serum concentrations were found elevated in 41 (89%) patients. Initial serum concentrations amounted to a median of 384 U l^−1^ (range 19–9578 U l^−1^).

**Figure 1:**
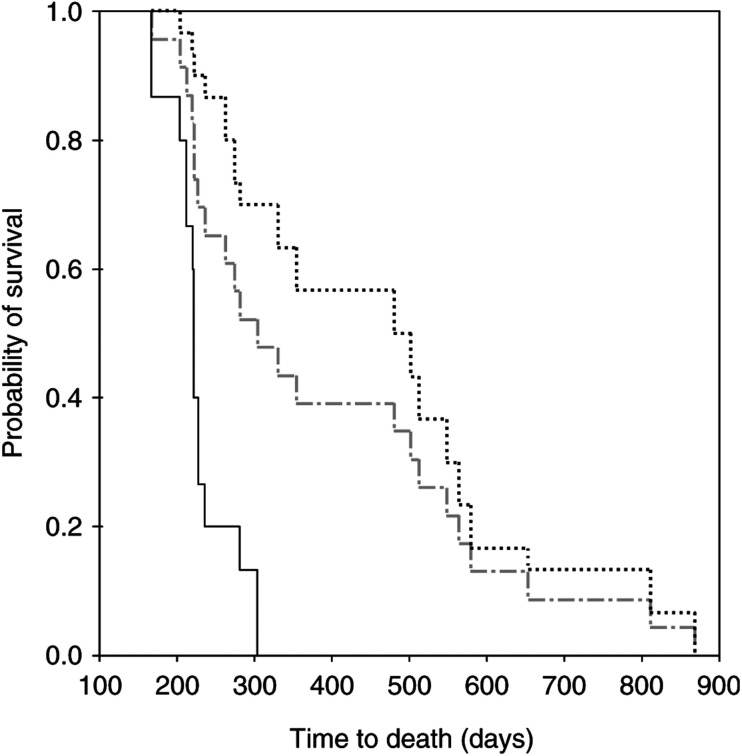
Survival curves comparing survival of patients treated with gemcitabine stratified according to CA 19-9 decrease <20% (**—**) and >20% (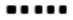) during 8 weeks of chemotherapy with gemcitabine. Line 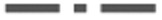 represents all patients.

The median survival of all patients was 392 days. The median survival of patients with normal CA 19-9 concentrations at baseline was 579 days (95% CI 442–734). Of the 41 patients, 13 (66%) had a biochemical response of CA 19-9 >20% after 8 weeks of chemotherapy to treatment with gemcitabine and 16 (34%) a progressive rise or a decrease of <20% (*n*=2) of the CA 19-9 concentration. The median overall survival of the 41 patients with increased baseline CA 19-9 concentrations was 281 days (95% CI 223–339 days). However, patients responding with CA 19-9 decrease >20% had a median survival of 383 days (95% CI 304–462 days), which was significantly longer than the median survival of the patients who did not respond (242 days, 95% CI 214–270 days; *P*=0.006, Table 1, Figure 1

). Patients responding with CA 19-9 >20% had a significantly longer median time to progression (199 days; 95% Cl 157–241 days) than patients who did not respond (61 days; 95% CI 33–89 days; *P*=0.002). Both the patients who achieved an objective complete response after 8 weeks of chemotherapy, had a CA 19-9 decrease >20%. Using a cutoff level of 20 U ml^−1^, none of the complete remission (CR) patients reached normal values in the course of treatment. In patients responding to chemotherapy with CR or partial remission (PR), imaging procedures like ultrasound, CT-scan, or MRT provided evidence of remission only after a median of 3.3 treatment courses (range=1–5 courses). All patients achieving PR showed a decrease of CA 19-9 >20% after 8 weeks, but none reached normal values.

Among patients with stable disease, 12 of the 19 patients and two of 20 with progressive disease after 8 weeks of treatment had decreasing CA 19-9 concentrations >20% and in the remaining 18 patients with progressive disease, CA 19-9 increased initially. This persisted >2 months in two. Trough levels of CA 19-9 were consistently above the cutoff level of 20 U ml^−1^. However, values increased later in all the 30 patients initially responding with the tumour marker CA 19-9 >20% and all of them experienced a recurrent increase. The median survival time from the beginning of recurrent rise to death was 220 days (95% Cl 177–262 days). Age and gender did not show significant differences or a trend for significant differences regarding survival by univariate analysis (Table 2

**Table 2:** Cox univariate and multivariate analyses of the prognostic factors and their impact on survival

	**Univariate analysis**		**Multivariate analysis**	
**Prognostic features**	***P*-value**	**HR (95% CI)^a^**	***P*-value**	**HR (95% CI)^a^**
*CA 19-9 response*	0.006		0.006	
>20%		1		1
<20%		0.23 (0.13–0.41)		0.26 (0.14–0.47)
				
*Clinical benefit response*	0.09			
pos.		1		
neg.		0.39 (0.27–0.56)		
				
*Age (years)*	0.35			
⩽60 (*n*=10)		1		
⩾60 (*n*=36)		0.56 (0.38–0.78)		
				
*Tumour grading*	0.72			
I/II		1		
III/IV		2.32 (0.70–7.69)		
				
*Tumour stage*	0.09			
III/IV A		1		
IV B		1.65 (1.12–2.42)		

a Hazard ratio (95% confidence interval) of univariate and multivariate analyses.

, *P*>0.05). Patients showing a clinical benefit response had a median survival of 506 days (95% CI 436–576 days), which was significantly longer than the median survival of the patients who did not respond (227 days, 95% CI 211–244 days; *P*=0.03). For the first CA 19-9 measurement after 3 weeks, we could not find such a relation.

In all, 22 patients experienced a decrease of pain, in nine patients the analgesic use was reduced, KPS was improved in 19 patients, and weight gain was observed in 12 patients. Response according to the response criteria of clinical benefit was seen in 12 patients. Two patients with objective complete response, three with objective partial response, and seven patients with objective stable disease experienced a clinical benefit response, whereas no patient with objective progressive disease had a clinical response according to the criteria. Two of three patients with objective partial response and clinical benefit response had a CA 19-9 decrease >20%, and of the 12 patients with objective stable disease and CA 19-9 decrease >20%, seven had a clinical benefit response.

## DISCUSSION

Patients with adenocarcinoma of the pancreas have a particularly poor survival with less than 1% alive 5 years from diagnosis. Recently, chemotherapy with gemcitabine has been shown for the first time to improve both survival and quality of life significantly in patients with advanced pancreatic cancer, although overall survival was still poor and the majority of patients treated did not respond (Burris *et al*, 1997). For the assessment of the objective response CT scan, ultrasound, or MRI are inaccurate (Rothenberg *et al*, 1996a). It has been proposed to be more appropriate to assess tumour response by clinical parameters and quality of life than assessment of tumour diameters. Tumour markers as biochemical response markers represent a potentially more simple and inexpensive method of monitoring response. The primary goal of this study was, therefore, to proof an early and reliable prognostic factor for remission evaluation.

In accordance with previous studies, the overall survival in our study was poor with none of the patients surviving longer than 29 months (Rothenberg *et al*, 1996b; Burris *et al*, 1997). We show that a significant correlation between objective treatment response and CA 19-9 serum concentrations at the start of treatment and, therefore, a decrease of CA 19-9 >20% after 8 weeks of chemotherapy is able to separate patients into groups with significantly different survival times. CA 19-9 changes not only at the beginning of the therapy but also a recurrent increase of CA 19-9 after an initial biochemical response was associated with a short median survival time of the patients. Unfavourable prognostic factors are the presence of metastatic disease, high preoperative concentrations of CA 19-9 (Lundin *et al*, 1994; Safi *et al*, 1998), and a poor performance status correlate with poor survival (Ishii *et al*, 1997). In our study, the alteration of CA 19-9 was the strongest independent prognostic factor, whereas clinical benefit response failed to be a prognostic factor. The time to measure a decrease of CA 19-9 ⩽20%, a rise during the first treatment courses of chemotherapy, or a recurrent rise after initial response is very short with 8 weeks; the measurement of CA 19-9 as a biochemical response marker after 8 weeks of chemotherapy may, together with clinical benefit response, help to decide whether further chemotherapy should be continued or stopped.

Patients' survivals with normal CA 19-9 at baseline were the longest. We can only speculate about this phenomenon. Tezel *et al* (2000) showed that pancreatic tumours with neuroendocrine-like differentiation showed a significantly better survival rate. In contrast to typical exocrine adenocarcinoma, neuroendocrine differentiation does not exhibit CA 19-9. We did not check neuroendocrine markers or the proportion of neuroendocrine differentiations in tissue samples, but we cannot exclude that a proportion of patients had increased neuroendocrine differentiation resulting in increased survival. No patient had decreasing CA 19-9 concentrations after 8 weeks of treatment despite disease progression. Different results were obtained in a phase II trial performing serial measurements in 14 patients with advanced pancreatic cancer treated with gemcitabine. All patients with radiologically assessed tumour (objective partial response *n*=2 or objective stable *n*=5), and also four of seven patients with progressive disease exhibited a decrease of CA 19-9 during the course of chemotherapy (Carmichael *et al*, 1996). Halm *et al* (2000) described two of seven patients with falling CA 19-9 concentrations despite disease progression. These inconsistent results remain difficult to interpret, but reflect the inaccuracies in the assessment of objective tumour size as described above.

The prognostic importance of CA 19-9 has already been established in patients undergoing radiation or resection for pancreatic cancer, specifically with regard to neoadjuvant treatment (Glenn *et al*, 1988; Lundin *et al*, 1994; Willett *et al*). Following radiotherapy, a better survival was found in patients responding with a falling CA 19-9 (Katz *et al*, 1998; Okusaka *et al*, 1998). On the contrary, increased CA 19-9 concentrations after resection of pancreatic cancer have been reported to be associated with poor survival compared to patients whose CA 19-9 had been normalised (Glenn *et al*, 1988; Tian *et al*, 1992; Safi *et al*, 1998). In this study, patients responding to chemotherapy with objective CRs or PRs demonstrated a fast and lasting reduction of CA 19-9. While the decline of CA 19-9 was observed already after the first treatment course, objective remissions were documented by imaging procedures only after a median of five chemotherapy courses. Only patients reaching objective CR also achieved normalisation of CA 19-9 levels. In patients in whom objective stable disease was determined by imaging techniques, CA 19-9 mostly declined demonstrating the biochemical efficacy of treatment without significant reduction of tumour volume.

A decrease of >15% of CA 19-9 concentrations during chemotherapy with 5-fluorouracil, epirubicin, and cisplatin has also been shown to correlate with a better survival and no objective progression as assessed by CT than a primary rise or a plateau of the CA 19-9 concentration (Gogas *et al*, 1998). The kinetics of CA 19-9 serum concentrations was prospectively evaluated in a selected group of patients treated with gemcitabine and cisplatin (Heinemann *et al*, 1999). The high remission rate (OR=38%) observed in this subgroup of patients certainly needs to be viewed critically. All patients received an objective complete response that demonstrated a significant decrease of CA 19-9 to normal values to the combined chemotherapy regimen. Also, similar to our study, patients with objective partial response or stable disease had significant tumour marker decrease. These results confirm that measurements of CA 19-9 help to assess prognosis during chemotherapy of pancreatic cancer.

Considering the limited number of patients in this study, data certainly need validation. However, the present results indicate that in patients with advanced or metastatic pancreatic cancer and increased baseline CA 19-9 concentrations, measurements of CA 19-9 and clinical benefit response should be performed after 8 weeks of treatment to assess prognosis together with other clinical parameters. Determination of CA 19-9 provides faster evidence of response than conventional imaging procedures. With decreasing CA 19-9 values, objective progress of tumour is improbable, and treatment may be continued. In patients with an increase of CA 19-9 or with a decrease ⩽20%, prognosis is extremely poor and with the exception of cases with significant improvement of clinical benefit response, further chemotherapy with gemcitabine seems to be of questionable value. In the setting of clinical decision-making, CA 19-9 kinetics may help to reduce the continuation of ineffective chemotherapy and the number of costly imaging procedures. Thus, rational implementation of CA 19-9 determination during chemotherapy of pancreatic cancer may induce a substantial reduction in treatment-related costs.
